# Cerebral Vasoreactivity Changes Over Time in Patients With Different Clinical Manifestations of Cerebral Small Vessel Disease

**DOI:** 10.3389/fnagi.2021.727832

**Published:** 2021-10-20

**Authors:** Jacek Staszewski, Aleksander Dȩbiec, Ewa Skrobowska, Adam Stȩpień

**Affiliations:** ^1^Military Institute of Medicine, Clinic of Neurology, Warsaw, Poland; ^2^Department of Radiology, Military Institute of Medicine, Warsaw, Poland

**Keywords:** neurovascular unit (NVU), cerebral small vessel disease, cerebrovascular reactivity (CVR), endothelial dysfunction, neurovascular coupling

## Abstract

**Objectives:** Endothelial dysfunction (ED) has been linked to the pathogenesis of cerebral small vessel disease (SVD). We aimed to assess ED and cerebrovascular reactivity (CVR) in the patients with a diverse manifestation of SVD, with similar and extensive white matter lesions (WMLs, modified Fazekas scale grade ≥2), compared with a control group (CG) without the MRI markers of SVD, matched for age, gender, hypertension, diabetes, and to evaluate the change of CVR following 24 months.

**Methods:** We repeatedly measured the vasomotor reactivity reserve (VMRr) and breath-holding index (BHI) of the middle cerebral artery (MCA) by the transcranial Doppler ultrasound (TCD) techniques in 60 subjects above 60 years with a history of lacunar stroke (LS), vascular dementia (VaD), or parkinsonism (VaP) (20 in each group), and in 20 individuals from a CG.

**Results:** The mean age, frequency of the main vascular risk factors, and sex distribution were similar in the patients with the SVD groups and a CG. The VMRr and the BHI were more severely impaired at baseline (respectively, 56.7 ± 18% and 0.82 ± 0.39) and at follow-up (respectively, 52.3 ± 16.7% and 0.71 ± 0.38) in the patients with SVD regardless of the clinical manifestations (ANOVA, *p* > 0.1) than in the CG (respectively, baseline VMRr 77.2 ± 15.6%, BHI 1.15 ± 0.47, *p* < 0.001; follow-up VMRr 74.3 ± 17.6%, BHI 1.11 ± 0.4, *p* < 0.001). All the assessed CVR measures (VMRr and BHI) significantly decreased over time in the subjects with SVD (Wilcoxon's signed-rank test *p* = 0.01), but this was not observed in the CG (*p* > 0.1) and the decrease of CVR measures was not related to the SVD radiological progression (*p* > 0.1).

**Conclusions:** This study provided evidence that the change in CVR measures is detectable over a 24-month period in patients with different clinical manifestations of SVD. Compared with the patients in CG with similar atherothrombotic risk factors, all the CVR measures (BMRr and BHI) significantly declined over time in the subjects with SVD. The reduction in CVR was not related to the SVD radiological progression.

## Background

Small vessel disease (SVD) is one of the most important cerebral microangiopathy, responsible for the majority of lacunar stroke (LS), vascular dementia (VaD), and parkinsonism (VaP) cases (Pantoni, 2010). The basic mechanism of the cerebral vessels alterations in the SVD is linked to endothelial dysfunction (ED), but whether ED only reflects the load of atherothrombotic risk factors or if it is specific to SVD has not been clearly defined (Forsberg et al., 2018). The neurovascular unit (NVU) concept accentuates the symbiotic association between brain cells, cerebral blood vessels, and subsequently cerebral blood flow (CBF) (Muoio et al., 2014). The endothelium and vascular smooth muscle within the NVU forms the basis of blood-brain-barrier (BBB), and contribute to the neurovascular coupling that is the response of the cerebral vessel to the changes in neural activity (Attwell et al., 2010). The SVD is a dynamic and progressive pathology involving variable components of NVU and BBB, however, the long-term clinical effects and outcomes usually differ between patients (Kisler et al., 2017). The recent studies have shown that the signaling pathways in the NVU control diverse processes, e.g., blood clotting and CBF, nevertheless it is not known precisely how CBF dysregulation translate to the disorders associated with neurovascular dysfunction, such as SVD. Cerebrovascular reactivity (CVR) is a measure of the capability of adaptive changes to vasodilatory stimuli (e.g., change in pCO_2_ due to CO_2_ inhalation, voluntary apnea, hyperventilation, or acetazolamide), and reflects the compensation of the collateral flow, therefore it can be used to indirectly assess and monitor the sequences of cerebral ED and the progression of vascular disease (Lavi et al., 2006). The reduced CVR indicates the impairment of NVU and regulatory mechanisms of CBF which result in neurovascular uncoupling. There are no biological markers to accurately assess brain vasoreactivity; however, CVR can be evaluated by various tools, such as single photon emission computed tomography (SPECT), PET, various MRI techniques, and transcranial Doppler ultrasound (TCD) (Terborg et al., 2000). Currently, one of the most widely used methods to measure the CVR is MRI, and it offers advantages over the use of radiolabeled products while maintaining regional specificity (Sleight et al., 2021). Though no direct anatomical information can be obtained, TCD permits for the evaluation of mean flow velocity (MFV) changes in the major cerebral arteries after a vasodilative stimulus and it provides complementary information of the brain hemodynamics (Ringelstein et al., 1988; Ebrahim et al., 2019; Burley et al., 2021a). The results of the TCD examination (MFV and pulsatility index) and the ventilation tests correlate with those obtained by other methods, and the value of TCD in the evaluation of CVR impairment, e.g., in the asymptomatic or symptomatic individuals with the brain white matter lesions (WMLs) has been established (Maeda et al., 1993; Marcos et al., 1997; Ghorbani et al., 2015; Fu et al., 2019). The assessment of CVR can be achieved with the bilateral recording of MFV in the middle cerebral artery (MCA) with acceptable reproducibility and inter-rater reliability using carbogen inhalation (McDonnell et al., 2013). Recently, we have found that the cerebral vasodilator responses to breath hold and hyperventilation were abnormal in the patients with VaD, LS, and VaP caused by SVD, and they were severely impaired when compared with the controls matched for the main vascular risk factors and free from the cerebrovascular events (Staszewski et al., 2019). Most of the studies assessing CVR in SVD did not evaluate the CVR changes over time or had unmatched control groups (CGs) (Thrippleton et al., 2018). Therefore, our study aimed to investigate the CVR changes over 24 months in the subjects with diverse manifestations of SVD (LS, VaD, and VaP), with similar and extensive radiological burdens of the disease and compare with a carefully selected CG without MRI markers of SVD, free of cerebrovascular events, and matched for major vascular comorbidities.

## Materials and Methods

### Participants

We analyzed the patients from the SHEF-CSVD Study in which the baseline and follow-up CVR and MRI imaging could be evaluated (Staszewski et al., 2013). The study protocol with detailed selection criteria and methodology has been described previously (Staszewski et al., 2019).

In brief: the SVD group consisted of ambulatory subjects above 60 years, enrolled between December 2011 and September 2015, with established LS (according to the OCSP criteria), VaP, or VaD (according to the Hurtig or NINDS-AIREN criteria) (Chui et al., 1992; Hurtig, 1993; Zijlmans et al., 2004). The patients with MRI contraindications, non-SVD-related WMLs, strategic single-infarct dementia, or post-stroke VaP or VaD, recurrent LS, carotid artery stenosis ≥50%, atrial fibrillation, chronic kidney disease (CKD) requiring dialysis, life expectancy <6 months, and recent head trauma were not included. To maximize the statistical power, we decided to analyze the equal groups of subjects and we recruited the consecutive patients with VaP and matched them in a 1:1 ratio with VaD, LS patients, and CG (without known cerebrovascular disease or dementia) according to sex, age (±5 years), and the presence of diabetes and hypertension. The signs of SVD are often seen in MRI in cognitively healthy elderly, and they are highly age-related. The studies showed that the WMLs are detected in 30–90% of cognitively healthy elderly (mean 72–74 years), therefore to maximize the homogeneity of the studied groups, we included only controls with no radiological signs of SVD in MRI (Fazekas 0) and the patients with SVD with extensive WMLs (Fazekas grade 2 or 3) (Longstreth et al., 2005; Gustavsson et al., 2015).

Of the 139 screened subjects (101 patients with SVD and 38 controls), 59 were excluded (18 controls, 15 LS, 9 VaD, and 17 VaP) due to inadequate acoustical bone window at the baseline (*n* = 16), the radiological markers of SVD in controls at baseline MRI examination (*n* = 3), withdrawal of consent during follow-up (*n* = 7), lack of follow-up MRI (*n* = 22), or TCD examination (*n* = 11). The patients with SVD and incomplete follow-up data (*n* = 30) had similar mean age (70.2 ± 7.6 vs. 72.6 ± 6.9, *p* = 0.14) and the baseline SVD score (2.1 ± 0.6 vs. 2 ± 0.6, *p* = 0.6) comparing with those included to the final analysis, however, more men dropped-out in comparison with women (48 vs. 9%, *p* < 0.01). The most common reported reason for lack of follow-up was that the subjects felt asymptomatic or did not tolerate well to the baseline MRI or TCD examination. Finally, the study group comprised 60 patients with newly diagnosed symptomatic SVD (20 per group: VaP, VaD, and LS) and 20 controls. All the patients were functionally independent (modified Rankin Scale, mRS ≤ 3 and total Barthel Index ≥ 80) and without severe dementia (Mini-Mental State Examination, MMSE ≥ 12) (Schulc et al., 2015).

### Study Procedures

All the patients signed informed consent before entering the study. The consent has been obtained prior to any study specific procedures. All the patients had TCD and MRI examinations performed at baseline and the 24-month follow-up visit (mean 23.1 ± 4 months; LS 22.5 ± 3; VaP 21.5 ± 4.6; VaD 23.3 ± 3.4; CG 23.6 ± 2; and ANOVA *p* = 0.28).

#### Ultrasound Examinations

The determination of the cerebrovascular reserve capacity is based on the ability of the intracranial arterioles to dilate. The CO_2_ tests assume the correlation among the CBF, the CO_2_ partial pressure, and the flow velocity in the basal cerebral arteries, and the reduction or elevation of pCO_2_ leads to a decrease or increase in MFV. Since TCD measures flow velocity in the large arteries, this reflects flow in the combined gray and white matter. The TCD study included evaluation of both the MCAs with 2-MHz probes (Companion III, Nicolet) in fixed positions according to the standard protocol (Settakis et al., 2002). The MFV values were averaged, and the interhemispheric differences for mean MFV did not exceed 15%. The recordings were considered acceptable when the velocities of blood flow could be detected bilaterally, and with a clear envelope of the MFV spectrum during the entire cardiac cycle. The CVR was measured as the breath-holding index (BHI), the ratio of the percentage MFV increase during hypercapnia, and vasomotor reactivity reserve (VMRr), the percentage change in MFV from hypo- to hypercapnia (Tsivgoulis and Alexandrov, 2008). Basing on the Markus and Harrison procedure, we measured the baseline MFV (baseMFV) following 10 min of rest with normal breathing (normocapnia), the minimal MFV value (minMFV) following 2 min of hyperventilation (hypocapnia), and the MFV value (maxMFV) subsequent to 30 s of breath holding (hypercapnia) and followed by a period of 4 min of normal breathing of room air (Markus and Harrison, 1992). To achieve the most reproducible results before proceeding to the definitive recording, the participants were trained to perform all the procedures correctly. All of them were able to hyperventilate and hold their breath for the required period. The exact length of breath-holding ranged from 29.8 to 30.5 s at the baseline and 30.1 to 30.3 s at follow-up, and it did not differ between the study groups (ANOVA, *p* > 0.1). The end-tidal CO_2_ (etCO_2_) concentration was monitored by capnograph (PC900A, Creative Medical, Shenzhen, China) during the examination. Blood pressure (BP) and heart rate (HR) were measured prior to and following the tests. Although McDonnell et al. found higher intra-rater reliability for the TCD measurements taken while the patients were sitting, we performed TCD examination in all the subjects in the supine position in accordance with our standard protocol (McDonnell et al., 2013). Ultrasound examination was performed under the standardized conditions (same quiet room and time of the day; no sleep deprivation and no medication intake for at least 6 h were allowed) by a single experienced TCD sonographer unaware of the diagnosis of the subjects. All the patients with LS had the study procedures performed at least 3 weeks (mean 24 ± 2 days) after their index strokes.

#### MRI Evaluation (GE Healthcare 1.5 T Scanner, IL, USA)

The images were evaluated for the presence of acute LS, lacunes, deep WMLs (dWMLs), or periventricular (pWMLs), microbleeds (MBs), and enlarged perivascular spaces (PVS) according to the STRIVE guidelines and visual SVD scale (Wardlaw et al., 2013; Staals et al., 2014). The simple modified Fazekas rating scale was used to estimate the extent of pWMLs and dWMLs (Fazekas et al., 1987; Inzitari et al., 2009). The mild white matter lesions (Fazekas grade 1) were defined as the punctate lesions in the deep white matter with a maximum diameter of 9 mm for every single lesion and 20 mm for grouped lesions. The moderate white matter lesions (grade 2) were defined as early confluent lesions of 10–20 mm for single lesions and >20 mm for grouped lesions in any diameter, with no more than connecting bridges between the individual lesions. Severe white matter lesions (grade 3) were defined as single lesions or confluent areas of hyperintensity of 20 mm or greater in any diameter. The presence of each of the four MRI markers for SVD (WMH, lacunes, cerebral microbleeds, and perivascular spaces) was counted to retrieve a total SVD score (ranging from 0 to 4). One point on the visual SVD scale was awarded if confluent deep WMLs (Fazekas grade 2 and 3) or irregular periventricular hyperintensities extending into the deep white matter (Fazekas grade 3) were present or when one or more lacunes or MBs were present; 1 point was awarded if moderate (10–25) to extensive (>25) enlarged PVS were present (Kim et al., 2008). The presence of each marker produced a score of a minimum 0 and a maximum of 4, representing the total MRI load of SVD. At baseline MRI assessment, all the patients with SVD had at least Fazekas grade 2 WMLs, and controls did not have radiological markers of SVD in MRI. The visual rating of SVD radiological progression expressed by the WMLs progression or development of new lacunes was performed at a follow-up visit. As proposed by Prins et al., the WMLs progression and lacunes were rated on the FLAIR images and the presence or absence of progression was rated in the three periventricular regions, basal ganglia, infratentorial region, and four subcortical white matter regions (Prins et al., 2004). The images were reviewed by a neuroradiologist (E.S.) blinded to the clinical data.

#### Atherothrombotic Risk Evaluation

The vascular comorbidities were defined according to the current standards and evaluated based on the available medical data, physical examinations, widespread histories, and routine laboratory tests performed at baseline (Alberti et al., 2009; Goblirsch et al., 2013).

### Statistical Analysis

All the demographic data were summarized, tabulated, and verified for normality with the Shapiro–Wilk test, and the homogeneity of the variances was assured by Levene's test. Categorical and continuous data are presented as frequencies or means ± SD and analyzed using Fisher's exact tests, the chi-square test, paired *t*-tests, or non-parametric tests where appropriate.

The associations between MRI progression with CVR changes over time in the studied groups were compared using a linear mixed model. The model included study group (SVD, CG), Time (Baseline, Follow-up), MRI (progression, no progression), and a Time^*^Group^*^MRI interaction for fixed effects. The subject variable was specified to assess possible individual variability. Per group, the Wilcoxon's signed-rank test was performed to evaluate the differences between CVR and other hemodynamic measures at the baseline and at follow-up visits. One-way ANOVA and chi-square tests were used to compare data between the study groups with *post-hoc* Tukey's honestly significant difference (HSD) tests for comparisons among the SVD subgroups. The effect sizes for the group differences were analyzed with partial eta squared ($\eta_{\text{p}}^{2}$) reflecting the proportion of the total variance attributable to the effect (and considered small if 0.01, moderate around 0.06, and high if >0.13). A probability value of *p* < 0.05 was considered significant, all the analyses were made using the PQStat 1.8.0 software.

The study has been approved by the Internal Revision Board (Wojskowy Instytut Medyczny, 46/WIM/2010) and it was conducted in accordance with the Declaration of Helsinki.

## Results

The studied cohort consisted of 80 older adults (mean 72.4 ± 6.2 years) of both sexes (50% women). The baseline characteristic of the studied cohort is provided in Table 1. The prevalence of the main comorbidities was similar in groups, but there were some differences in the laboratory examination findings: the CG had lower concentrations of homocysteine, showed a trend toward lower concentration of uric acid, and had higher levels of estimated glomerular filtration rate (eGFRs) comparing with other subjects. The SVD subgroups had a similar prevalence of vascular risk factors and treatment use, laboratory, and MRI findings (Fazekas grade 3 was scored by 80% LS, 80% VaD, and 75% VaP subjects; *p* = 0.7). The mean etCO_2_ concentrations and systolic blood pressure (SBP), diastolic blood pressure (DBP), and heart rate (HR) measures during hyperventilation and breath holding did not differ between the CG and the SVD group, and they were also similar between the SVD subgroups. In addition, these variables did not significantly differ between the baseline and follow-up assessments (Wilcoxon's sign rank test, *p* > 0.1). The reproducibility measurements of VMRr and BHI were performed in a sample of 12 individuals (four from CG, eight with LS). The intraclass correlation coefficient for the two consecutive measurements was 0.90; *p* < 0.01 for VMRr and 0.88; *p* < 0.01 for BHI.

**Table 1:** Main characteristics of the study population at baseline.

**Variable**	**SVD (*n =* 60)**	**LS (*n =* 20)**	**VaP (*n =* 20)**	**VaD (*n =* 20)**	**CG (*n =* 20)**	***p* ^#^**
**Demographics**						
Age (y)^*^	72.6 (±6.9)	71.7(±7.7)	73.8 (±6.2)	72.2 (±6.8)	71.9 (±3.2)	0.7
Female gender (%)^*^	30 (50)	10 (50)	10 (50)	10 (50)	10 (50)	1
**Vascular risk factors**						
Hypertension (%)^*^	54 (89)	18 (90)	18 (90)	18 (90)	18 (90)	1
Diabetes (%)^*^	30 (50)	10 (50)	10 (50)	10 (50)	10 (50)	1
CAD (%)	22 (37)	8 (40)	7 (35)	7 (35)	7 (35)	0.2
Current smoking (%)	22 (37)	7 (35)	6 (30)	9 (45)	8 (40)	0.5
Hyperlipidemia (%)	45 (75)	15 (75)	16 (80)	14 (70)	15 (75)	0.8
CKD (%)	8 (13)	2 (10)	2 (10)	4 (20)	3 (15)	0.3
PS (%)	28 (46)	10 (50)	10 (50)	8 (40)	8 (40)	0.8
Obesity (BMI>30)	17 (28)	6 (30)	6 (30)	5 (25)	6 (30)	0.8
**Examination findings**						
Homocysteine (μmol/l)	14.4 ± 6.1	13.2 ± 3.7	18.1 ± 8.3	12.03 ± 3.5	13.1 ± 2.7	0.01
hsCRP (mg/dL)	0.55 ± 0.9	0.56 ± 1.02	0.64 ± 0.9	0.46 ± 0.8	0.16 ± 0.17	0.2
Uric acid (mg/dL)	5.4 ± 1.5	5.8 ± 1.6	5.5 ± 1.3	4.8 ± 1.5	4.6 ± 1.3	0.05
eGFR (ml/min/1.73 m^2^)	78.8 ± 24.1	77.1 ± 29	72.3 ± 22.8	87 ± 17.2	95 ± 16	0.01
HbA1c (%)	6.2 ± 1.03	6.3 ± 1.2	5.9 ± 0.5	6.3 ± 1.2	5.9 ± 0.1	0.26
FG (mg/dL)	120.1 ± 43.2	128.8 ± 48.6	112.8 ± 32.3	120.6 ± 47.2	106.3 ± 18.7	0.21
LDL (mg/dL)	108.4 ± 37.8	97.1 ± 35.5	110.6 ± 36.6	116.5 ± 40.4	122.8 ± 41.8	0.22
HDL (mg/dL)	52.2 ± 17	46.7 ± 10	55.2 ± 16.9	54.1 ± 22.5	60.9 ± 19.3	0.12
TG (mg/dL)	124.5 ± 57.9	125.6 ± 60.1	132.1 ± 64.2	115.5 ± 50	115.1 ± 39	0.7
TC (mg/dL)	185.2 ± 48.1	166.8 ± 39.5	194.4 ± 54.7	193.4 ± 54.7	198.7 ± 31.7	0.1
BMI	26.4 ± 5.6	27.7 ± 7.6	25.1 ± 4	26.3 ± 4.5	27.8 ± 3.8	0.3
**MRI examination**						
SVD score (mean ± SD)	2 ± 0.6	2.2 ± 0.7	2 ± 0.6	2.1 ± 0.6	0	<0.01
Fazekas pWMLs (mean ± SD)	1.45 ± 0.9	1.5 ± 1.1	1.4 ± 1	1.5 ± 0.7	0	<0.01
Fazekas dWMLs (mean ± SD)	1.6 ± 0.9	1.7 ± 1.1	1.5 ± 0.8	1.7 ± 0.9	0	<0.01
**Vascular treatment**						
Statin treatment (%)	45 (75)	15 (75)	16 (80)	14 (70)	15 (75)	0.8
ACEI (%)	15 (25)	6 (30)	4 (20)	5 (25)	7 (35)	0.6
Beta blocker (%)	22 (37)	8 (40)	7 (35)	7 (35)	7 (35)	0.2
ARB (%)	19 (32)	6 (30)	7 (35)	6 (30)	7 (35)	0.4
Calcium channel blocker (%)	23 (38)	9 (40)	7 (35)	7 (35)	7 (35)	0.4
Diuretics (%)	12 (20)	4 (20)	3 (15)	5 (25)	4 (20)	0.2

*Values represent the means (±SD) for continuously distributed data or the numbers (%) for categorical data*.

* *Matched factors*;

# *ANOVA and χ^2^ difference between the groups p <0.05 compared with CG according to Tukey's honestly significant difference (HSD) test*.

*SVD, cerebral small vessel disease; LS, lacunar stroke; VaD, vascular dementia; VaP, vascular parkinsonism; CG, control group; CAD, coronary artery disease; BMI, body mass index; FG, fasting glucose; PS, polymetabolic syndrome; CKD, chronic kidney disease; IMT, intima-media thickness; ACEI, angiotensin-converting-enzyme inhibitors, ARB, angiotensin receptor blockers*.

Vasomotor reactivity reserve and the BHI were more severely impaired at the baseline and at follow-up assessments in the patients in the SVD groups (regardless of the clinical spectrum of the disease) than in the CG (Table 2). The size effect of the difference was similar in all comparisons (eta-squared ranged between 0.25 and 0.29). For better visualization, the CVR change in the individual subjects is shown in (Supplementary Figures 1, 2).

**Table 2:** Basal and follow-up characteristics and vasodilatation responses of cerebral arteries in all the subjects.

**Variable**	**SVD (*n =* 60)**	**LS (*n =* 20)**	**VaP (*n =* 20)**	**VaD (*n =* 20)**	**CG (*n =* 20)**	***p* ^#^**
**Baseline measures**						
MFV (cm/s)	41.5 ± 12^*^	43.9 ± 15	42 ± 9.5	38.4 ± 10.4^*^	46.8 ± 8.7	0.03
VMRr (%)	56.7 ± 18.4^*^	55.4 ± 18.6^*^	55.1 ± 15.2^*^	54.2 ± 18.7^*^	77.2 ± 15.6	<0.001
BHI	0.82 ± 0.39^*^	0.86 ± 0.4^*^	0.77 ± 0.28^*^	0.71 ± 0.4^*^	1.15 ± 0.47	<0.001
Resting SBP, mmHg	127.2 ± 17.2	130.1 ± 16.2	126.1 ± 11.1	125.2 ± 16	124 ± 19	0.2
Resting DBP, mmHg	73.4 ± 10.1	72.4 ± 13.2	73.2 ± 12.4	74.4 ± 11	74.1 ± 8	0.7
Resting HR, beats/min	76.8 ± 6.6	76.8 ± 4.7	75.2 ± 8.2	78.3 ± 6.6	72.1 ± 6.1	0.8
Delta etCO_2_ HV (%)	1.3 ± 0.15	1.4 ± 0.4	1.3 ± 0.2	1.1 ± 0.7	1.4 ± 0.5	0.6
Delta etCO_2_ BH (%)	1.2 ± 0.1	1.3 ± 0.2	1.3 ± 0.6	1.1 ± 0.6	1.2 ± 0.4	0.8
**Follow-up measures**						
MFV (cm/sek)	35.2 ± 11.2^*^	37.1 ± 15.6	34.3 ± 9.2^*^	34.2 ± 8.3^*^	43.1 ± 8.2	0.06
VMRr (%)	52.3 ± 16.7^*^	54.4 ± 17.9^*^	49.3 ± 11.5^*^	53.2 ± 19.7^*^	74.3 ± 17.6^*^	<0.001
BHI	0.71 ± 0.38^*^	0.72 ± 0.5^*^	0.71 ± 0.24^*^	0.69 ± 0.4^*^	1.11 ± 0.4^*^	<0.001
Resting SBP, mmHg	124.8 ± 13.9	127.2 ± 17.1	125.3 ± 9.8	122.1 ± 15	127 ± 9	0.3
Resting DBP, mmHg	76.3 ± 11	74.1 ± 14.1	78.1 ± 7.7	76.7 ± 11	74.2 ± 10	0.6
Resting HR, beats/min	73.9 ± 2.2	72.6 ± 3	75.2 ± 1.2	74.1 ± 2.4	70 ± 10.5	0.2
Delta etCO_2_ HV (%)	1.23 ± 0.6	1.3 ± 0.7	1.2 ± 0.5	1.2 ± 0.6	1.9 ± 0.7	0.3
Delta etCO_2_ BH (%)	1.16 ± 0.7	1.1 ± 0.8	1.1 ± 0.4	1.3 ± 0.4	1.2 ± 0.7	0.8

# *ANOVA difference between the groups*,

* *p < 0.05 compared with the CG*.

*SVD, cerebral small vessel disease; LS, lacunar stroke; VaD, vascular dementia; VaP, vascular parkinsonism; CG, control group; MFV, mean flow velocity; BHI, breath holding index; VMRr, vasomotor reactivity reserve; PI, pulsatile index; RI, resistance index; SBP, systolic blood pressure; DBP, diastolic blood pressure; HR, heart rate*.

Δ *etCO_2_HV*, Δ *end-tidal CO_2_ during hyperventilation*; Δ *etCO_2_ BH*, Δ *end-tidal CO_2_ during breath holding*.

Vasomotor reactivity reserve and the BHI were significantly lower in diabetic patients with SVD vs. diabetic controls at the two timepoints (baseline VMRr: 55.8 ± 16 vs. 74 ± 18%, respectively, *p* < 0.001; BHI: 0.79 ± 0.39 vs. 1.1 ± 0.3, *p* = 0.02; follow-up VMRr 52.7 ± 15 vs. 71.2 ± 20, *p* < 0.01; BHI: 0.72 ± 0.4 vs. 1.04 ± 0.5, *p* < 0.01 and the difference was also significant between the non-diabetic subjects from SVD group vs. CG (respectively; baseline VMRr: 54.2 ± 18.4 vs. 79.9 ± 13.7, *p* < 0.001; BHI: 0.78 ± 0.39 vs. 1.16 ± 0.55, *p* = 0.01; follow-up VMRr 52.1 ± 18 vs. 76.8 ± 14.9%, *p* < 0.01; BHI: 0.70 ± 0.35 vs. 1.15 ± 0.2; *p* < 0.001).

**Figure 1:**
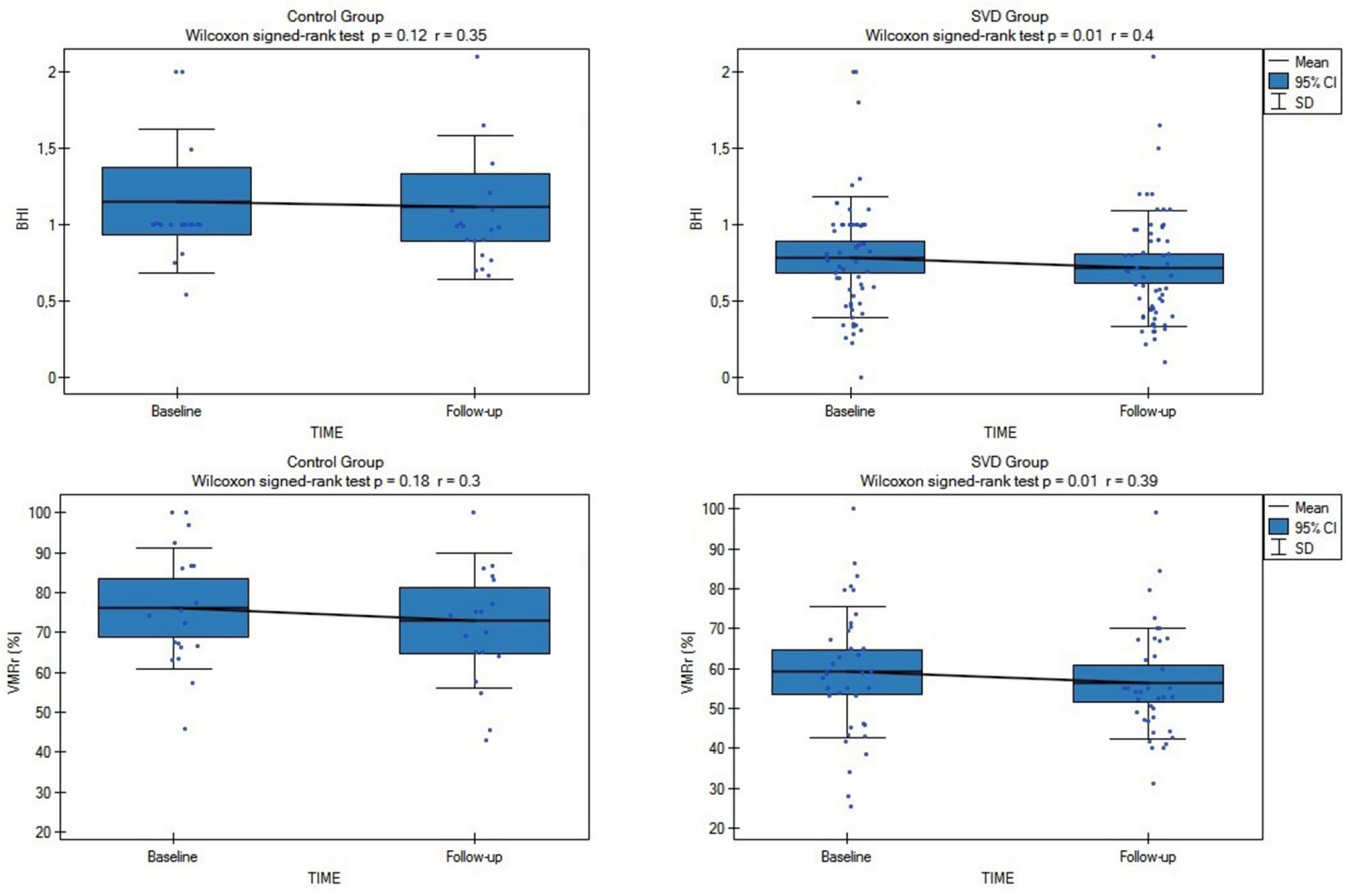
Comparison of baseline and follow-up cerebrovascular reactivity (CVR) measures (VMRr [vasomotor reactivity reserve] and breath-holding index [BHI]) in all subjects from cerebral small vessel disease (SVD) and control group (CG). SVD, cerebral small vessel disease; VMRr, vasomotor reactivity reserve; BHI, breath-holding index.

Both the assessed CVR measures (VMRr and the BHI) significantly declined over time in the subjects with SVD (Wilcoxon's signed-rank test, *p* = 0.01), however, there was no significant decline of VMRr and BHI in CG over 24 months of observation (Wilcoxon's signed-rank test *p* > 0.1) (Figure 1). The mean VMRr and BHI values were also similar at the baseline and follow-up measurements among the patients with LS, VaD, and VaP (ANOVA, *p* > 0.1).

**Figure 2:**
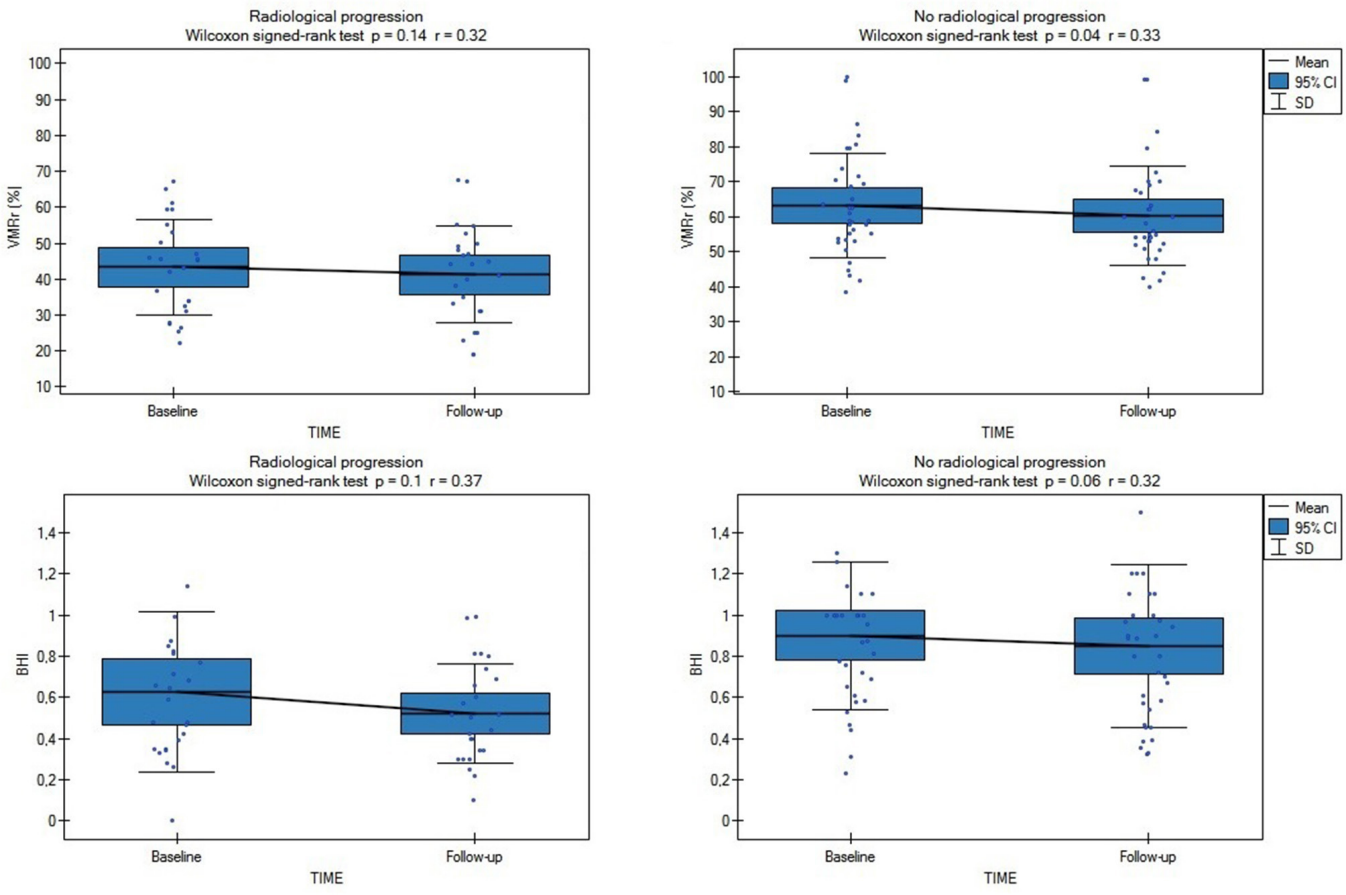
Comparison of baseline and follow-up CVR measures (VMRr and BHI) in SVD subjects with (*n* = 25) and without radiological progression (*n* = 35). VMRr, vasomotor reactivity reserve; BHI, breath-holding index.

Radiological progression during the study was observed in 28 of the 80 subjects (35%): significantly more often (*p* = 0.03) in the SVD subjects (*n* = 25 [42%]: eight subjects from LS [40%] group, eight with VaD [40%], and nine with VaP [45%], with no difference between the SVD groups [ANOVA *p* > 0.1]) than in the CG (*n* = 3; 15%). During the follow-up, only 4/80 patients (5%) experienced lacunar stroke (two patients from LS group and two from VaD group), no other vascular events were observed. Although in patients with SVD who experienced radiological progression, all the CVR measures were considerably impaired at the baseline and follow-up assessments compared with the no progression group (respectively, baseline VMRr 43.3 ± 13.2% vs. 63.3 ± 14.9%, *p* < 0.001; eta-square 0.33; baseline BHI 0.63 ± 0.39 vs. 0.9 ± 0.36, *p* < 0.001; eta-square 0.12; follow-up VMRr 41.2 ± 13.4% vs. 60.3 ± 14%, *p* < 0.001; eta-square 0.32; BHI 0.52 ± 0.24 vs. 0.84 ± 0.39, *p* < 0.001; eta-square 0.19), no significant decrease of CVR measures was observed during 24 months of observation (Wilcoxon's signed-rank test, *p* > 0.1) (Figure 2). These data did not change significantly when we analyzed all the patients (SVD and CG) with the radiological progression vs. no progression group (respectively, baseline VMRr 45.4 ± 14.1% vs. 68.6 ± 16.9%, *p* < 0.001; baseline BHI 0.65 ± 0.37 vs. 1.0 ± 0.42, *p* < 0.001; follow-up VMRr 43.1 ± 14.3% vs. 65.7 ± 16.9%, *p* < 0.001; BHI 0.55 ± 0.24 vs. 1.0 ± 0.47, *p* < 0.001) and there was also no significant decrease of CVR measures over time of observation (Wilcoxon's signed-rank test, *p* > 0.1). No interaction between CVR measures, time of assessment, and radiological progression in the SVD group were observed in the fixed linear analysis (Table 3).

**Table 3:** Association among the cerebral small vessel disease (SVD), assessment time point, and MRI progression on the cerebrovascular reactivity (CVR) measures.

						**95% CI**	
**Dependent variable**	**Fixed factor(s)**	**B**	**SE**	***t***	*P* ^*^	**Lower limit**	**Upper limit**
**BHI**							
	Time	−0.01	0.05	−0.01	0.90	−0.11	0.11
	SVD	−0.47	0.11	−4.26	<0.01	−0.69	−0.25
	MRI progression	−0.60	0.22	−2.70	0.01	−1.04	−0.16
	Time^*^SVD	0.05	0.07	0.55	0.50	−0.20	0.38
	Time^*^MRI progression	0.08	0.18	1.20	0.24	−0.15	0.59
	SVD^*^MRI progression	0.29	0.24	1.23	0.22	−0.18	0.77
	Time^*^SVD^*^MRI progression	−0.02	0.16	−0.13	0.80	−0.34	0.30
**VMRr**							
	Time	−3.90	2.60	−1.40	0.20	−9.20	1.30
	SVD	−27.10	4.34	−6.20	<0.01	−35.70	−18.50
	MR progression	−28.80	9.20	−3.10	0.01	−47.10	−10.50
	Time^*^SVD	7.05	3.23	2.13	0.20	−0.20	5.97
	Time^*^MRI progression	9.04	6.80	1.32	0.20	−4.50	22.60
	SVD^*^MRI progression	9.80	9.90	0.90	0.30	−9.98	29.60
	Time^*^SVD^*^MRI progression	−10.07	7.40	−1.35	0.17	−24.80	6.70

* *Linear mixed effects model*.

*BHI, breath holding index; VMRr, vasoreactivity reserve; SVD, small vessel disease*.

## Discussion

This prospective study performed in symptomatic SVD has shown that the change in CVR measures is detectable following a 24-month period and that 42% of patients with SVD demonstrated radiological progression. We also confirmed that the cerebral vasoreactivity measures decreased in SVD regardless of the clinical manifestation of the disease. On the opposite, we did not demonstrate a significant CVR decrease in the subjects from CG who were neurologically asymptomatic, had no radiological markers of SVD in the baseline MRI, but who shared similar atherothrombotic risk factors as the SVD group. Although patients with SVD and radiological progression had severely impaired CVR at the baseline and follow-up comparing with the subjects with no MRI progression, there was no significant interaction between that CVR decline and risk of radiological progression.

Besides of growing evidence of MRI techniques which increasingly play a more important role as a non-invasive tool capable to assess the cerebral reserve capacity in combination with a vascular challenge, the vasomotor reactivity testing with TCD is still a gold diagnostic standard and it has been proved to assess CBF and indirectly monitor the function of ED in patients with cerebral microangiopathy (Kozera et al., 2009; D'Andrea et al., 2016). The TCD studies demonstrated that CVR is negatively correlated with the duration of hypertension, patient age, and history of cerebrovascular events (Fujishima et al., 2001; Kozera et al., 2010). Some older studies using SPECT or Xenon CT techniques revealed that LS, particularly with leukoaraiosis was associated with the reduced CBF in the white matter and cortex in the subjects with severe WMLs, while the studies on VaD consistently showed reductions in both the white and gray matter (Markus et al., 2000). Many studies investigated CBF in the patients with SVD using TCD techniques, and in line with our data, the majority of them revealed impaired CBF comparing with the healthy controls in the patients with symptomatic and asymptomatic LS, more marked in multiple comparing with single infarcts and a reduction in reactivity that was correlated with the degree of leukoaraiosis (Table 4) (Maeda et al., 1993; Molina et al., 1999; Terborg et al., 2000). There is, however, a lack of studies on the dynamic CVR changes over time, especially in SVD, and in well-characterized SVD populations, e.g., in rarely studied patients with VaD and VaP. As SVD is a wide term and it is used to describe the different disease processes ranging from asymptomatic WMLs in normal individuals through to the symptomatic SVD subjects, as we aimed in the presented report, our data added some further evidence that cerebral ED occurs and progresses in patients with LS, VaD, or VaP due to SVD, even if they had well-controlled hypertension (according to the baseline and follow-up BP measurements). Interestingly, a similar trend was not observed in CG with comparable atherothrombotic risk factors (age, sex, diabetes, hypertension, obesity, and smoking), and this may suggest that other vascular factors or duration of exposure to vascular risk factors in SVD are more important and responsible for a continuous decline in the CVR (Muoio et al., 2014). For example, the studies on patients with cerebrovascular disease showed that the reduction in vasodilatory capacity in asymptomatic carotid disease can predispose for the development of cerebrovascular disease (Silvestrini et al., 2000). In 2020, Soman et al. (2020) using arterial spin labeling MRI to evaluate which cardiovascular risk factors alter the CVR found that higher SBP, chronic kidney disease, history of past stroke, and hypercholesterolemia are responsible for the impaired CVR. Although the controls and patients with SVD in our study were well-balanced with the main comorbidities, we did not control other important risk factors of SVD. For instance, chronic poor sleep patterns, depression, prediabetes, and unhealthy diet (dietary salt) can impact the endothelial function through immune influences (Hakim, 2019). Of notice, the Oxford Vascular (OXVASC) Study importantly showed that premorbid hypertension in midlife correlated more strongly with the global SVD burden than a single blood pressure measurement or a known history of hypertension (Lau et al., 2018).

**Table 4:** Major transcranial Doppler ultrasound (TCD) studies assessing CVR in sporadic small vessel disease populations.

**References**	**Study population**	**CVR assessment**	**Results**
Molina et al. (1999)	46 patients with lacunar stroke and 46 CG	Acetazolamide test	Significant CVR reduction in SVD group
Terborg et al. (2000)	46 patients with SVD and 13 CG	Ventilation tests; NIRS	Significantly reduced VMR in TCD and NIRS assessments in severe SVD; a good correlation between the validity of TCD and NIRS
Kidwell et al. (2001)	55 patients with SVD	PI	Correlation between PI and severity of SVD ; PI cut points allowed discrimination of PVH with 89% sensitivity and 86% specificity and discrimination of DWMH with 70% sensitivity and 73% specificity.
Pánczél et al. (2002)	25 patients with lacunar stroke, 20 patients with leukoaraiosis and 35 CG	Tilting, ventilation, and acetazolamide tests	CVR in BA and MCA territory was impaired in hypercapnia in SVD; no significant differences between CVR measures in BA and MCA territory in acetazolamide test; significantly higher VRCO_2_ in MCA vs BA measurements
Ghorbani et al. (2015)	56 patients with SVD and 48 CG	PI	Good correlation between PI and SVD radiological manifestations: - In PVH with PI cut-off = 0.83, the sensitivity 90% and specificity 98% - In DWMH with PI = 0.79, the sensitivity 75% specificity 87.5% - In lacunar stroke with PI = 0.80, the sensitivity 73% and specificity 90%. - In PH with PI = 0.69, the sensitivity 92% and specificity 87.5%. - In PVH+ DWMH+ lacunar with PI = 0.83, the sensitivity 90% and specificity 96%.
Nam et al. (2020)	206 patients with lacunar stroke	PI	PI was positively associated with the WMHs volume and the presence of old lacunar infarcts.

*TCD, transcranial Doppler ultrasound; PI, pulsatile index; MRI, magnetic resonance imaging; SVD, cerebral small vessel disease; CG, control group; PVH, preventricular hyperintensity; DWMH, deep white matter hyperintensity; CVR, cerebrovascular reactivity; BA, basilar artery; MCA, middle cerebral artery; VRCO, ventilatory response to CO_2_; PH, pontin hyperintensity; NIRS, near-infrared spectroscopy; CT, computed tomography*.

Whether the progressive decrease of CVR is specific to the progressive nature of SVD is unknown (Stevenson et al., 2010). Importantly, we showed that there was no difference in the CVR at the baseline and follow-up assessments between the SVD clinical subgroups and this finding possibly could be related to similar WMLs burden or comparable prevalence of comorbidities. We also observed that the subjects with radiological progression had the most impaired CVR at baseline which, however, did not significantly decrease over time. These findings may indicate that MRI progression in that group may be related to the baseline severe CVR impairment or other factors, e.g., inflammatory or prothrombotic. It is also possible that the TCD measures are less sensitive to the more subtle changes, particularly in the white matter flow. Our results are, however, opposite to a large study in 628 asymptomatic individuals (mean 69 years) with WMLs which showed a progressive reduction in the CBF velocity measured using TCD as WMLs severity increased (Tzourio et al., 2001). Some similar data were demonstrated by Sam et al., who established areas of reduced CVR that preceded the development of WMLs by advanced MRI techniques, suggesting that CVR impairment contributes to the development and progression of SVD (Sam et al., 2016). On the opposite, in a PROSPER trial of statin therapy that investigated 390 individuals with cardiovascular risk factors, there was no association between CBF and the prevalence of WMLs at baseline, a decline of CBF, and risk of development of deep WMLs (Ten Dam et al., 2007). Our results are also in line with the study that showed significantly reduced CVR in LS than in the CG, which, however, could not be explained by the main atherothrombotic risk factors (Deplanque et al., 2013). It is important to appreciate that cerebral reactivity and autoregulation are not fully understood, although the neurogenic, metabolic, or myogenic factors were proposed. Of notice, CO_2_-CVR is only one of the several mechanisms of cerebral autoregulation, thus preserved CO_2_-CVR does not imply intact cerebral autoregulation (Klinzing et al., 2021). Therefore, CVR impairment could be due to different mechanisms, e.g., related to genetic factors or BBB dysfunction, resulting in microvascular dysfunction (Zlokovic, 2011). Some important aspects of the present study related to the patients with VaD and VaP should be underlined as these subjects probably share SVD pathology with neurodegenerative disease, and it is not established whether the microvascular changes reflect the diminished demand caused by the advanced neurodegeneration or whether SVD precedes and contributes to the neurodegeneration. For example, impaired cerebrovascular reserve capacity and vasoreactivity (identified using TCD along with the BHI) was found in the patients with mild cognitive impairment, Alzheimer's disease, and Parkinson's disease without severe underlying atherosclerosis (Shim et al., 2015; Urbanova et al., 2018). Although the contribution of CVR impairment to the pathogenesis of neurodegenerative diseases is not certain, it might be suspected that a reduced cerebrovascular reserve is an additional deteriorating factor in the neurodegeneration (Ojeda et al., 2017). One unanswered key question is that whether any reductions in the CBF are primary, or merely occurs secondary to the brain damage as due to vaso-neuronal coupling, the reductions in brain metabolism are associated with reduced CBF (Markus et al., 2014).

Most studies in the literature have demonstrated the differences in CBF between the groups of patients with SVD and controls, with very few studies addressing the sensitivity and specificity of these parameters in TCD and MRI examinations (Panerai, 2009). The large variations in CVR across the patients and between different sessions of the same subject are often observed in the TCD and MRI studies, which hamper the ability of these measures in monitoring disease progression (Hou et al., 2020). It should be especially stressed that there are concerns with the application of the BHI as the relationship between breath-hold length and the pCO_2_ stimulus remains unclear and this test has low reproducibility and high variability (Alwatban et al., 2018; Koep et al., 2020). The latest studies showed that the blood oxygenation level dependent MRI (BOLD-MRI) method is most reliable and reproducible because it has the advantage of mapping the whole brain with good spatial resolution, allowing investigation of CVR regional distribution (Leoni et al., 2012). Despite the relationships between the baseline CBF measures from TCD and MRI, the current studies showed no direct correlations between the CVR metrics calculated from TCD and MRI-BOLD measures during a 5%CO_2_ challenge (Burley et al., 2021b). These variations in the CVR measures in different imaging modalities are important as they significantly reduce the statistical power to detect the pathology-related differences, preclude personalized determination of abnormalities, and challenge the validity of comparing CVR metrics across the studies.

Our study has several limitations, and the data should be cautiously interpreted owing to the single-center design and a small number of subjects included that may introduce bias. Even if we analyzed a relatively homogeneous cohort and highly selected SVD sample of patients, it should be considered a hypothesis-generating pilot study, because it may be less representative of the overall SVD population. However, the majority of the TCD studies on CVR in subjects with SVD had low patient numbers or controls (Table 4). Although the patients were prohibited from taking any drugs before the examinations, some medications could have affected the dilatory responses of the arteries. In future studies, additional research may be needed to determine the effect of treatment as a function of CBF on the prognosis of patients. The other limitations are that we used 1.5 Tesla MRI which potentially limited the accuracy of the radiological assessment, and we did not account for etCO_2_ for calculating the CVR. This could have influenced the results and limit the generalizability and reproducibility of the study, since PaCO_2_ has a marked influence on CBF and autoregulation, evaluations could be compromised in situations where significant changes in PaCO_2_ are undetected (Panerai et al., 1999). However, in our study, no significant difference between the subgroups and between the baseline and follow-up etCO_2_ measures were noticed. Despite the multitude of alternatives to the gas challenges, existing literature lacks definitive conclusions regarding the best practices for the vasoactive modulation and associated analysis protocols for the TCD studies resulting in numerous metrics of cerebral autoregulation (Valdueza et al., 2008; Sanders et al., 2018). Regardless of methodology, an assessment of cerebrovascular autoregulation is prone to moderate noise and artifact, with low reliability and reproducibility (Lee et al., 2020). In our study, CVR was assessed using TCD which additionally suffers from a limited field of view comparing with the MRI techniques and thus it may be less reflective of the local changes in the tissue blood supply. However, due to limited access and the high cost of MRI examination, the non-invasive, bedside, and acceptable repeatable assessments using TCD, it remains still the most utilized tool to study the CBF regulation in humans. As different techniques have low correlations and target different parts of the vascular tree, they should, however, be regarded as complementary and they are recommended to be used together (Purkayastha and Sorond, 2012; Burley et al., 2021b). Another important limiting factor is that the hemodynamic effect of breath holding is lower than that of CO_2_ inhalation or acetazolamide injection, therefore the validity of our results should be confirmed in the future (Marcic et al., 2021). The TCD measurements are limited to the large basal arteries and can only provide an index of global rather than local CBF velocity, and it is highly operator dependent. However, all the TCD tests in our study were carried out by a single certified examiner who has 15 years of experience working with TCD, and thus we have minimized a possible interpersonal difference depending on the experience of the examiner.

The present study has, however, some important strengths. To the best of our knowledge, no data have been published on repeatedly assessed CVR measures over 24 months of observation in a well-phenotyped cohort of patients with SVD with extensive radiological markers of SVD. Ours is the first study to investigate all CVR by TCD in a wide range of participants, such as normal controls and patients with LS, VaD, and VaP. Our findings of impaired and progressive CVR alterations in the SVD subjects are important as this mechanism could contribute to exacerbating the clinical condition and may constitute a potential line of research for the treatment. A better understanding of the variations in CVR is of importance in both clinical and basic science applications (Hou et al., 2020). The majority of data on CVR are derived from the studies on individuals with asymptomatic WMLs, therefore, our study, which included homogenous groups of symptomatic SVD subjects, is important (Blair et al., 2016).

In conclusion, we provided further evidence that cerebral ED occurs and progresses over time in the patients with different clinical manifestations of SVD, however, we did not observe a significant CVR difference between the subjects with SVD and radiological progression comparing with no progression group and no significant CVR alterations over time in the CG who were neurologically asymptomatic and who shared similar comorbidities to the SVD group. The longitudinal studies with larger sample sizes are needed to definite the causal conclusions and confirm these findings.

## Data Availability Statement

The raw data supporting the conclusions of this article will be made available by the authors, without undue reservation.

## Ethics Statement

The studies involving human participants were reviewed and approved by Local Medical Ethics Committee, Military Institute of Medicine, Warsaw, Poland (46/WIM/2010). The patients/participants provided their written informed consent to participate in this study.

## Author Contributions

JS: conceptualization, methodology, validation, data curation, and project administration. JS and ES: investigation. JS and AD: writing—original draft preparation. JS and AS: supervision. All authors have read and agreed to the published version of the manuscript.

## Funding

This study was supported by the Polish Ministry of Science and Higher Education as a research project of the Military Institute of Medicine (Warsaw, Poland, study number N N402 473840).

## Conflict of Interest

The authors declare that the research was conducted in the absence of any commercial or financial relationships that could be construed as a potential conflict of interest.

## Publisher's Note

All claims expressed in this article are solely those of the authors and do not necessarily represent those of their affiliated organizations, or those of the publisher, the editors and the reviewers. Any product that may be evaluated in this article, or claim that may be made by its manufacturer, is not guaranteed or endorsed by the publisher.
