# Supplementary figures and images for: Cerebral Vasoreactivity Changes Over Time in Patients With Different Clinical Manifestations of Cerebral Small Vessel Disease
Source: Front Aging Neurosci. 2021 Oct 20;13:727832. doi: 10.3389/fnagi.2021.727832 (PMC8563577; doi:10.3389/fnagi.2021.727832)

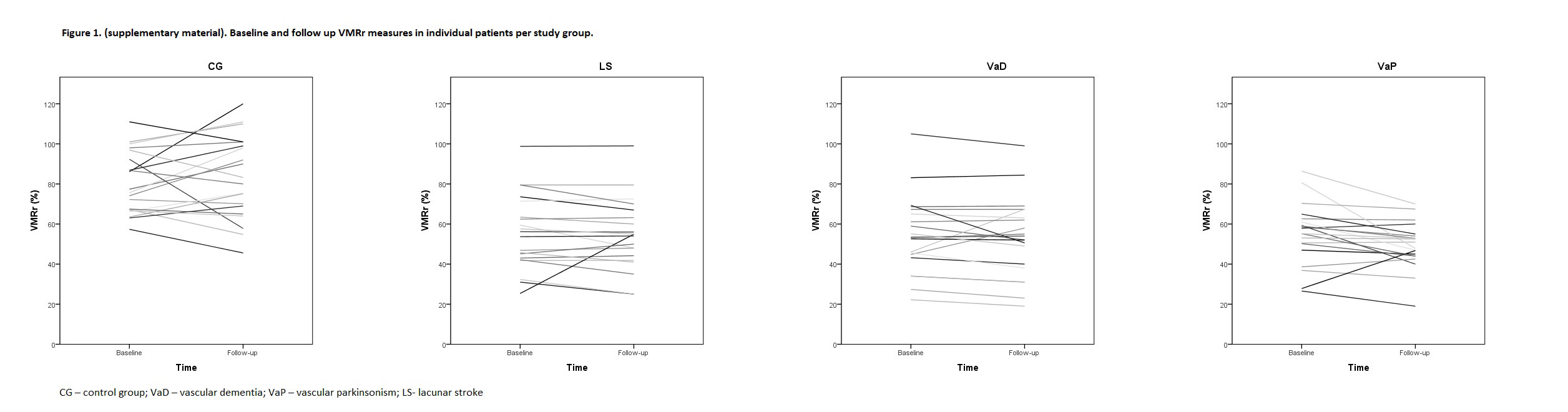

Supplement: Supplementary file 1 [file Image_1.JPEG]

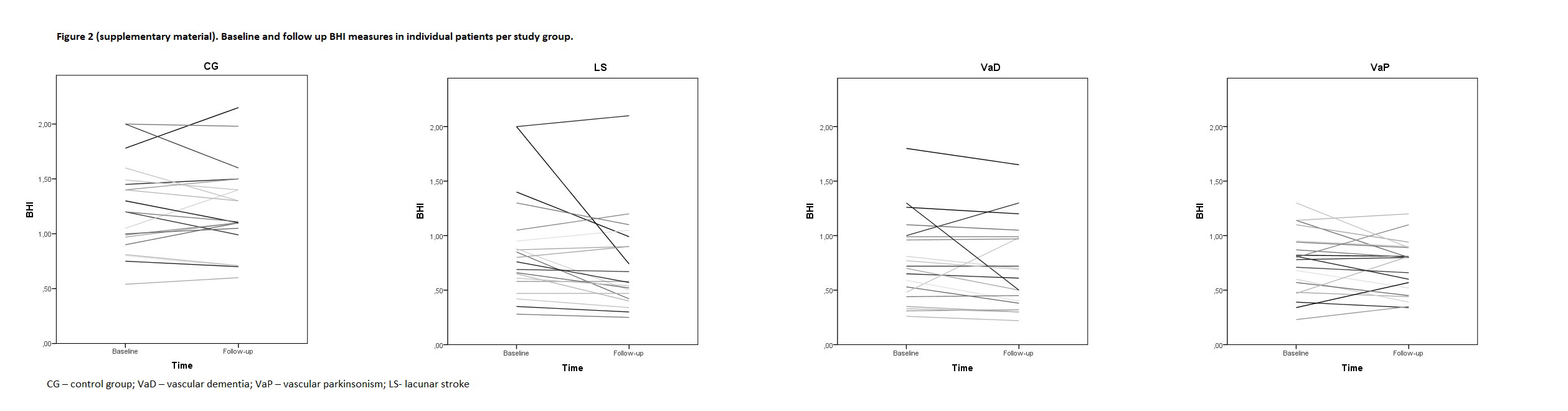

Supplement: Supplementary file 2 [file Image_2.JPEG]
