# Supplementary material for: Cerebral Vasoreactivity Changes Over Time in Patients With Different Clinical Manifestations of Cerebral Small Vessel Disease
Source: Front Aging Neurosci. 2021 Oct 20;13:727832. doi: 10.3389/fnagi.2021.727832 (PMC8563577; doi:10.3389/fnagi.2021.727832)
Supplement: Supplementary file 3 [file Table_1.DOCX]

**Table S1 (supplementary material). MRI sequence parameters (1.5T GE Healthcare MR scanner). The same sequences were used at presentation and at 24 months follow-up.**

| Sequence | T1W spin echo | DWI/DTI  (30 diffusion directions) | FLAIR (TI=2200 ms) | T2W Fast spin echo | T2*W gradient recalled echo (FA=20^o^) |
| --- | --- | --- | --- | --- | --- |
| Orientation | Sagittal | Axial | Axial | Axial | Axial |
| TE (ms) | 10 | 109 | 120 | 65 | 15 |
| TR (ms) | 640 | 6000 | 8000 | 5400 | 1020 |
| FOV | 26 x 26 | 26 x 26 | 26 x 26 | 26 x 26 | 24 (AP) x 18 |
| Slice thickness (mm) | 3.0 | 5.0 | 3.0 | 3.0 | 3.0 |
| Slice gap (mm) | 0 | 0 | 0 | 0 | 0 |
| Matrix | 512(SI) x 160 | 128 x 128 | 320 x 224 | 384 x 192 | 384(AP) x 224 |
| No. slices | 51 | 54 | 51 | 51 | 50 |
